# Traditional Herbal Medicine and Allergic Asthma

**DOI:** 10.1155/2015/510989

**Published:** 2015-04-28

**Authors:** Bi-Fong Lin, Bor-Luen Chiang, Yan Ma, Jin-Yuarn Lin, Miaw-Ling Chen

**Affiliations:** ^1^Department of Biochemical Science and Technology, National Taiwan University, Taipei 10617, Taiwan; ^2^Graduate Institute of Immunology, National Taiwan University, Taipei 10048, Taiwan; ^3^Department of Pathophysiology and Allergy Research, Center of Pathophysiology, Infectiology & Immunology, Vienna General Hospital, Medical University of Vienna, Vienna, Austria; ^4^Department of Food Science and Biotechnology, National Chung Hsing University, Taichung 40227, Taiwan; ^5^Department of Nutrition and Health Science, Chang Jung Christian University, Tainan City 71101, Taiwan

The frequency of allergic diseases such as asthma and allergic rhinitis has increased rapidly during the past decade; however, the exact mechanisms have still not been established. Both air pollution and change of diet habit have been thought to play an important role in increasing prevalence of atopic diseases. Atopic diseases were mediated predominantly by type 2 T helper- (Th2-) mediated activity including allergen-specific IgE antibody and eosinophils. Allergic asthma is a chronic disease with the characteristics of immune response mediated by type 2 T helper- (Th2-) related cytokines and IgE antibody. Allergic airway inflammation has been characterized by the infiltration of Th2 cells and eosinophils, subsequently followed by the bronchial constriction and mucus secretion.

Conventional treatments for allergic asthma include steroids, leukotriene antagonists, bronchodilators, and most recent anti-IgE antibody. All these drugs are still with certain shortcomings such as side effects, effectiveness, and cost. It has become more and more important to develop novel therapeutic approaches for the treatment of allergic asthma. Complementary medical approaches such Chinese herb medication and acupuncture have been suggested to play a role in the immune regulation of diseases [1]. More studies have focused on exploring the possibility of these complementary therapeutic approaches for the treatment of immunological diseases [2]. All these complimentary therapeutic approaches have been regarded as having less side effects and being used as the adjuvant therapy for the diseases [3]. Furthermore, many researchers also aim to identify the active components of herb medicine for the purification and development of drugs.

In this special issue, we have five articles including the survey of traditional Chinese medicine application for the treatment of allergic asthma and also the study on the traditional Tianjiu therapy for the treatment of asthmatic patients. Furthermore, three articles on studying the active components of herb medicine have been included in the issue. S.-I. Lin et al. analyzed the use of Chinese traditional medicine for the treatment of allergic asthma in Taiwan. They collected 20,800 newly diagnosed asthmatic children and found out 20% of them actually used traditional Chinese medicine as the treatment of their asthma. In addition, they also analyzed the most frequent used herbal medicine for allergic asthma in the article.

A variety of components purified from medicinal herbs have been found to exert the immune modulatory effect on many diseases [4]. Among the components, polyphenols, triterpenoids, and polysaccharides are found to be the most effective in the anti-inflammatory or immune modulation of the diseases. M.-L. Chen et al. have identified both triterpenoids and polysaccharide portion for the treatment of allergic diseases. The results showed that triterpenoids portion of* Ganoderma tsugae* exerted anti-inflammatory activity and polysaccharide portion had the immune stimulatory effect instead. C.-M. Ku and J.-Y. Lin also identified an active component of farnesol, a sesquiterpene alcohol, exerting anti-inflammatory activity and alleviating airway inflammation in murine model of asthma. M.-L. Chen et al. also identified ethanol extract of* Perilla frutescens* which also exerted anti-inflammatory activity and also alleviated allergic airway inflammation. Finally, L. Zhu et al. studied the effect of Tianjiu therapy in Sanfu Days for the treatment of asthmatic children. The results suggested that Tianjiu therapy could decrease the dose of bronchodilator during the asthma attack. However, the symptoms of allergic asthma did not show significant improvement after treatment. More studies are needed for the application of Tianjiu therapy for the treatment of allergic asthma in the future.

The prevalence of clinical immunological diseases such as autoimmune diseases and allergic diseases is increasing in recent years. More and more efforts have been dedicated to develop novel therapeutic approaches for the treatment of these immune-mediated diseases. Complimentary medicine such as herb drugs or acupuncture has been suggested to be useful for the treatment of a variety of clinical immunological diseases [5]. Many of these herb medicinal components have been found to exert anti-inflammatory activity and modulation of immune response. Although more studies are needed to identify the novel compounds and the treatment, it might still be the future target for the novel therapeutic development. Particularly, these herb medicinal drugs are noted with fewer side effects, which might also be useful for possible adjuvant therapy.

*Bi-Fong Lin* *Bi-Fong Lin* *Bor-Luen Chiang* *Bor-Luen Chiang* *Yan Ma* *Yan Ma* *Jin-Yuarn Lin* *Jin-Yuarn Lin* *Miaw-Ling Chen* *Miaw-Ling Chen*
